# A Novel Imaging Biomarker for Cerebral Small Vessel Disease Associated With Cognitive Impairment: The Deep-Medullary-Veins Score

**DOI:** 10.3389/fnagi.2021.720481

**Published:** 2021-10-25

**Authors:** Zhihua Xu, Fangfei Li, Dengxiang Xing, Hongyan Song, Jingshu Chen, Yang Duan, Benqiang Yang

**Affiliations:** ^1^Department of Radiology, Tongde Hospital of Zhejiang Province, Hangzhou, China; ^2^Center for Neuroimaging, Department of Radiology, General Hospital of Northern Theater Command, Shenyang, China; ^3^General Hospital of Northern Theater Command Training Base for Graduate, Dalian Medical University, Shenyang, China; ^4^Center for Medical Data, General Hospital of Northern Theater Command, Shenyang, China; ^5^General Hospital of Northern Theater Command Training Base for Graduate, Jinzhou Medical University, Shenyang, China; ^6^General Hospital of Northern Theater Command Training Base for Graduate, China Medical University, Shenyang, China; ^7^Department of Radiology, General Hospital of Northern Theater Command, Shenyang, China

**Keywords:** deep medullary veins, cerebral small vessel disease, cognitive impairment, aging, total CSVD score CSVD and DMVs

## Abstract

**Objective:** To explore the biomarkers of cerebral small vessel disease (CSVD) associated with cognitive impairment.

**Methods:** A total of 69 patients with CSVD were enrolled in the study, and baseline clinical and imaging data were reviewed retrospectively. The following neuroimaging biomarkers of CSVD were identified: high-grade white matter hyperintensity (HWMH), cerebral microbleeds (CMB), enlarged perivascular space (PVS), and lacunar infarct (LI). A total score for CSVD was calculated. The deep medullary veins (DMVs) were divided into six segments according to the regional anatomy. The total DMV score (0–18) was derived from the sum of the scores of the six individual segments, the scores of which ranged from 0 to 3, for a semiquantitative assessment of the DMV that was based on segmental continuity and visibility.

**Results:** The DMV score, patient age, and total CSVD score were independently associated with the presence or absence of cognitive impairment in patients with CSVD (*P* < 0.05). By integrating patient age and the total CSVD and DMV scores, the area under the curve of the receiver operating characteristic curve (AUROC) for predicting CSVD associated with cognitive impairment was 0.885, and the sensitivity and specificity were 64.71 and 94.23%, respectively.

**Conclusions:** The DMV score may be a novel imaging biomarker for CSVD associated with cognitive impairment. The integration of the DMV score with age and total CSVD score should increase the predictive value of the DMV score for CSVD associated with cognitive impairment.

## Introduction

Cerebral small vessel disease (CSVD) is common and is one of the main causes of cognitive impairment in the elderly (Chen et al., 2019). Magnetic resonance imaging (MRI) is a useful approach for the assessment of CSVD. The neuroimaging biomarkers of CSVD (Wardlaw et al., 2013) include the following: white matter hyperintensity (WMH), cerebral microbleeds (CMBs), enlarged perivascular spaces (PVS), and lacunar infarcts (LIs). A CSVD score that integrates these four radiological biomarkers can not only indicate the severity of CSVD but also predict the risk of cognitive decline and dementia (Banerjee et al., 2018; Pavlovic et al., 2018; Jiang et al., 2019; Liu et al., 2019; Amin Al Olama et al., 2020). However, each neuroimaging biomarker of CSVD and the total CSVD score only consider the abnormalities seen on MRI. They are not a direct reflection of the histopathological changes occurring at the level of the small blood vessels.

A few recent studies have found that changes in the deep medullary veins (DMVs) seen on susceptibility-weighted imaging (SWI), a unique MRI technique that visualizes cerebral veins *in vivo*, were associated with the presence of CSVD and total CSVD score (Zhang et al., 2017; Chen et al., 2020; Xu et al., 2020; Zhou et al., 2020). These findings may enable us to increase our understanding and exploration of the histopathological changes leading to CSVD. However, in these recent studies, the relationship between the changes in the DMVs to cognitive impairment in patients with CSVD was not comprehensively observed. Thus, we aimed to assess the DMVs visualized on SWI as a novel marker for evaluating CSVD associated with cognitive impairment.

## Materials and Methods

### Patients

**FIGURE 1:**
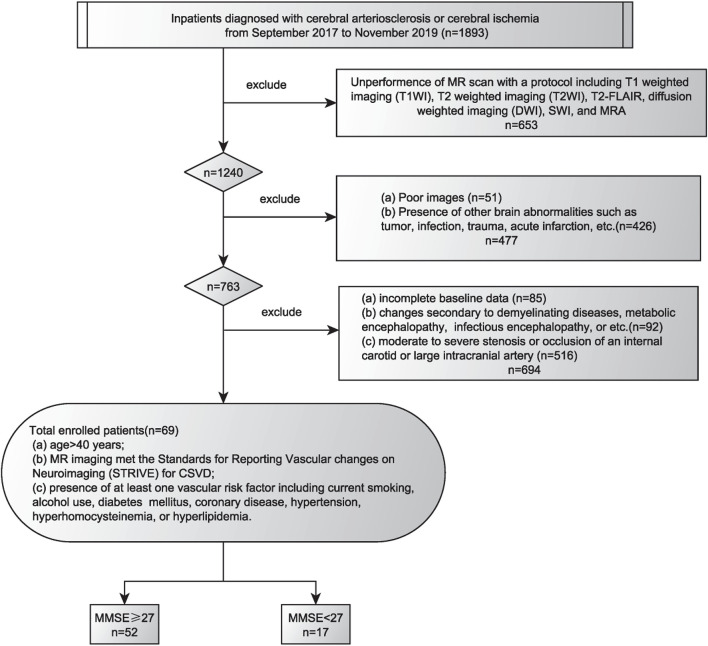
Flowchart of the enrollment of study patients. MMSE, Mini-Mental State Examination score.

The protocol for this study was approved by the Institutional Review Board of the General Hospital of the Northern Theater Command. Each patient or his/her legally authorized representatives provided written informed consent prior to participation in this study. The clinical and imaging data of patients with CSVD were collected and reviewed from September 2017 to November 2019. The inclusion criteria were as follows: (a) age > 40 years; (b) education years > 6; (c) underwent the following MRI protocols, including T1-weighted imaging (T1WI), T2-weighted imaging (T2WI), T2-FLAIR, diffusion-weighted imaging (DWI), SWI, and MR angiography (A); (d) MRI reports satisfied the standards for reporting vascular changes on neuroimaging (STRIVE) for CSVD (Wardlaw et al., 2013); and (e) patient had at least one cerebrovascular risk factor, including current smoking, alcohol use, diabetes mellitus, coronary artery disease, hypertension, hyperhomocysteinemia, or hyperlipidemia. The exclusion criteria were as follows: (a) incomplete baseline data; (b) changes secondary to demyelinating diseases, including metabolic encephalopathy or infectious encephalopathy; (c) presence of other brain abnormalities such as tumor, infection, trauma, and acute infarction; (d) moderate to severe stenosis or occlusion of an internal carotid or large intracranial artery; (e) patients with hereditary CSVD; and (f) another disease associated with cognitive impairment, such as Alzheimer disease. The flowchart showing enrollment of the study patients is shown in Figure 1.

### Clinical Information

The baseline information of the study patients was collected from the medical records and included the following: sex; age; C-reactive protein and risk factors for CSVD, including current smoking, alcohol use, diabetes mellitus, coronary disease, hypertension, blood pressure (systolic, diastolic, mm HG), hyperhomocystinemia, and hyperlipidemia. The Mini-Mental State Examination (MMSE) score was used to evaluate the cognitive impairment. An MMSE score of less than 27 was used to identify patients with cognitive impairment (Delavaran et al., 2017).

### Magnetic Resonance Imaging Protocol

All patients underwent multimodal MRI on a 3.0 T Discovery MR750 scanner (General Electric Healthcare, Chicago, IL, United States) equipped with an eight-channel phased-array head coil. The settings used were as follows:(1) SWI: repetition time (TR) = 27 ms; echo time (TE) = 20 ms; flip angle = 10°; slice thickness = 2 mm; intersection gap = 0 mm; field of view (FOV) = 24 × 24 cm^2^; resolution = 0.5 mm × 0.5 mm in plane, with 96 slices, acquisition matrix of 320 × 224 and a total scan time of 4 min; k-space points were zero-filled to 512 × 512; (2) T2-FLAIR: TR = 8,800 ms; TE = 94 ms; inversion time = 2,500 ms; slice thickness = 5 mm; intersection gap = 1 mm; FOV = 24 × 24 cm^2^; resolution = 0.5 mm × 0.5 mm in plane, with acquisition matrix of 320 × 160; k-space points were zero-filled to 512 × 512.

### Total Score for Cerebral Small Vessel Disease

All images were reviewed separately by two neuroradiologists. Disagreements were resolved by consensus. We identified WMH, CMB, PVS, and LI according to the STRIVE criteria (Wardlaw et al., 2013) to estimate the total CSVD score. WMH was defined as abnormal hyperintensity of the periventricular white matter or deep white matter on T2-FLAIR images. The Fazekas scoring system was used to estimate the extent of WMH. The presence of high-grade (H)WMH was considered to be a Fazekas score of ≥2 in the periventricular white matter and/or ≥2 in the deep white matter. CMBs were defined as homogeneous hypointensities with a mean diameter ranging from 3 to 5 mm on SWI after excluding calcification, cross section of a vessel, and abnormal iron deposits. An enlarged PVS was defined by small dot-like or linear fluid signals accompanied by small blood vessels on MR images. The presence of high-grade (H)PVS was identified by finding more than 10 enlarged PVS’s at the level of the maximum number of PVS’s in the unilateral basal ganglia. LIs were defined as round or ovoid subcortical lesions of 3–15 mm in diameter that manifested as hyperintense lesions on T2WI and as hypointense lesions on T1WI. Finally, the total CSVD score was based on an ordinal scale ranging from 0 to 4, depending on the absence or presence (0 or 1) of each of the four features of CSVD (HWMH, CMB, HPVS, and LI).

### Deep Medullary Veins Score

**FIGURE 2:**
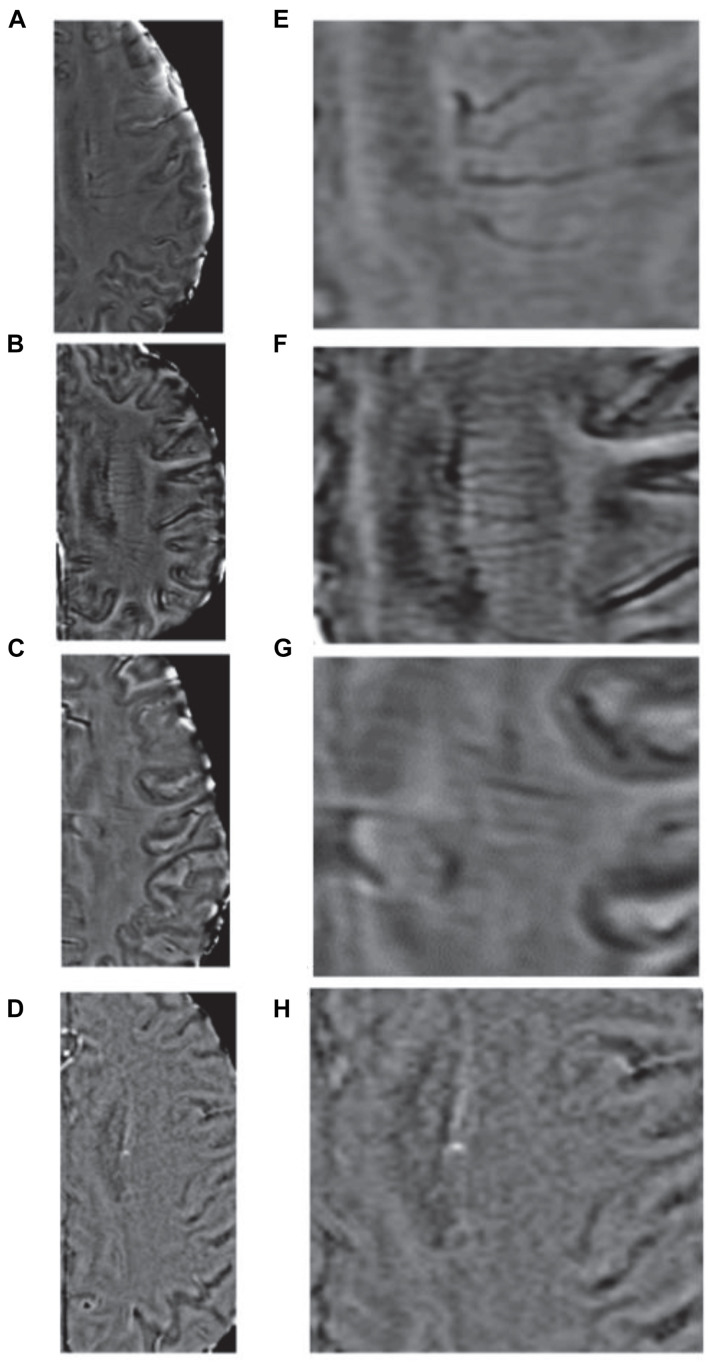
Deep medullary veins scoring system. **(A,E)** A score of 0 is assigned for each continuous and prominently visible vein; **(B,F)** A score of 1 indicates that the vein is continuous and with unequivocal visibility, but at least one vein manifests an inhomogeneous signal; **(C,G)** A score of 2 indicates that at least one vein is not continuous and is faintly visible, presenting spot-like hypointensities; **(D,H)** A score of 3 is assigned if no DMVs are visible.

Deep medullary vein scores were determined on the SWI sequences. We assessed DMVs on five consecutive periventricular slices (10-mm thickness) of SWI phase images from the level of the ventricles immediately above the basal ganglia to the level of the ventricles immediately disappeared for each patient, with the assumption that these slices would contain most of the DMVs. The bilateral regions containing the DMVs were divided into six segments, the frontal, parietal, and occipital segments. For the semiquantitative assessments of the DMVs, each segment was scored separately based on its continuity and visibility, with DMV scores ranging from 0 to 3 (Zhang et al., 2017). A DMV score of 0 indicated a clearly visible, continuous segment with no interruptions. A score of 1 indicated clearly visible, continuous veins in the segment, but with an inhomogeneous signal from at least one vein. A score of 2 indicated at least 1 faintly visible, discontinuous vein manifesting spot-like hypointensity. A score of 3 indicated the absence of DMVs (Figure 2). The total DMV score consisted of the sum of DMV scores from the six segments and ranged from 0 to 18. Thus, a score of 0 indicated prominent DMVs, while a score of 18 indicated no obvious DMVs seen. All images were reviewed separately by two neuroradiologists who were completely blinded to the patients’ clinical data and extent of CSVD.

### Statistical Analysis

Categorical variables are reported as frequencies and percentages; normally distributed continuous data are reported as means and standard deviations (SD); and non-parametric data are reported as medians and interquartile ranges (IQRs). Differences between normally distributed data were analyzed by the *t*-test; differences between categorical variables were analyzed by the chi-squared test; and differences between non-parametric data were analyzed by the Mann–Whitney *U* test or Kruskal–Wallis test. Univariate analysis was performed to compare the baseline clinical and imaging characteristics of patients stratified according to presence and absence of cognitive impairment. Binary logistic regression analysis was then performed to identify independent factors associated with the presence of cognitive impairment in patients with CSVD. Receiver operating characteristic (ROC) curve analysis was performed to evaluate the diagnostic value of neuroimaging biomarkers for predicting CSVD associated with cognitive impairment. The Youden index was used to determine the sensitivities and specificities. Pairwise comparison of area under the receiver operating curves (AUCs) was performed using the Delong’s test (DeLong et al., 1988) between different model to determine whether the difference was statistically significant. Statistical significance was considered to be *P* < 0.05. Data were analyzed by the Statistical Package for Social Sciences for Windows, Version 20 (IBM Corp., Armonk, NY, United States).

## Results

This study evaluated 69 patients with CSVD (47 males). Their mean age was 66 ± 11 years. Among the 69 patients, HWMH was seen in 40 (58.0%), CMB in 42 (60.9%), HPVS in 42 (60.9%), and LI in 43 (62.3%). The median (IQR) CSVD and DMV scores were 2 (1, 4) and 8 (6, 11) respectively. MMSE scores less than 27 were seen in 17 patients with cognitive impairment.

### Inter-Reader Agreement for the Evaluations of DMV and CSVD Burden

Images of 51 patients were excluded because of poor quality. The quality of MRI images of enrolled patients satisfied the diagnostic standards and allowed the neuroradiologists to identify DMV and each neuroimaging CSVD biomarker. The agreement between readers was excellent for DMV scores of SWI (κ = 0.842) and for the CSVD burden on T2-FLAIR images (κ = 0.921).

### Factors Related to CSVD Associated With Cognitive Impairment

The relationships between cognitive impairment and the radiological and clinical features of patients with CSVD are shown in Tables 1, 2. Univariate analysis identified the following characteristics that were associated with the presence or absence of cognitive impairment in patients with CSVD: age, periventricular WMHs, LIs, enlarged PVS’s, burden of CSVD, and DMV score. However, the differences between the following characteristics of patients with/without CSVD were not significant: sex, current smoking, alcohol use, diabetes mellitus, coronary disease, degree of hypertension, blood pressure (systolic, diastolic, mm HG), C-reactive protein, hyperhomocystinemia, and hyperlipidemia.

**TABLE 1:** Baseline clinical characteristics and comparisons between study patients stratified by presence or absence of cognitive impairment.

	Cognitive impairment		
	No (*n* = 52)	Yes (*n* = 17)	*P*-value
Age	63 ± 9	75 ± 12	< 0.001
Male	36 (69.2)	11 (64.7)	0.962
Education year	11 ± 3	10 ± 3	0.150
MMSE	30 (28,30)	25 (18,26)	< 0.001
Current smoking	18 (34.6)	6 (35.3)	1.000
Alcohol use	15 (28.8)	6 (35.3)	0.843
Diabetes mellitus	27 (51.9)	12 (70.6)	0.286
Coronary disease	11 (21.2)	6 (35.3)	0.395
**Hypertension**			0.115
Grade 0	8 (15.4)	0 (0.0)	
Grade 1	12 (23.1)	7 (41.2)	
Grade 2	14 (26.9)	2 (11.8)	
Grade 3	18 (34.6)	8 (47.1)	
**Blood pressure**			
Systolic, mm HG	149 (135, 163)	153 (143, 171)	0.179
Diastolic, mm HG	88 (79, 97)	90 (83, 97)	0.712
C-reactive protein	1.60 (0.78, 3.57)	1.40 (0.70, 2.40)	0.780
Hyperhomocystinemia	13.65 (10.38, 18.52)	13.60 (11.80, 17.10)	0.867
Hyperlipidemia	15 (28.8)	6 (35.3)	0.843

**TABLE 2:** Imaging characteristics of study patients grouped by the presence or absence of cognitive impairment.

	Cognitive impairment		
	No (*n* = 52)	Yes (*n* = 17)	*P-*value
CMB	30 (57.7)	12 (70.6)	0.510
WMH	2 (1, 3)	3 (2, 6)	0.007
Periventricular WMH	1.00 (1, 2)	2.00 (2, 3)	< 0.001
Deep WMH	0.00 (0, 2)	2.00 (0, 3)	0.073
Lacunar infarcts	28 (53.8)	15 (88.2)	0.024
PVS	26 (50.0)	16 (94.1)	0.003
Total CSVD	2 (1, 3)	3 (2, 4)	0.004
DMV score	7 (5, 10)	12 (9, 14)	0.001

*CSVD, cerebral small vessel disease; WMH, white matter hyperintensity; CMB, cerebral microbleed; PVS, perivascular spaces; DMV, deep medullary veins.*

To avoid interactions between the total CSVD score and other neuroimaging biomarkers of CSVD, we just selected the total CSVD score when a binary logistic regression analysis was performed. The DMV score, patient age, and total CSVD score were found to be independently associated with the presence or absence of cognitive impairment in patients with CSVD (*P* < 0.05, Table 3).

**TABLE 3:** Multivariate analysis of the related factors of cognitive impairment in patients with cerebral small vessel disease.

	OR	OR value (95% C.I.)		*P*-value
		Lowest	Highest	
Age	1.102	1.022	1.189	0.011
Burden of CSVD	2.03	1.107	3.21	0.022
DMV score	1.339	1.091	1.794	0.008

*CSVD, cerebral small vessel disease; DMV, deep medullary veins.*

### Diagnostic Characteristics of the Factors Identified to Be Independently Associated CSVD-Associated Cognitive Impairment

**FIGURE 3:**
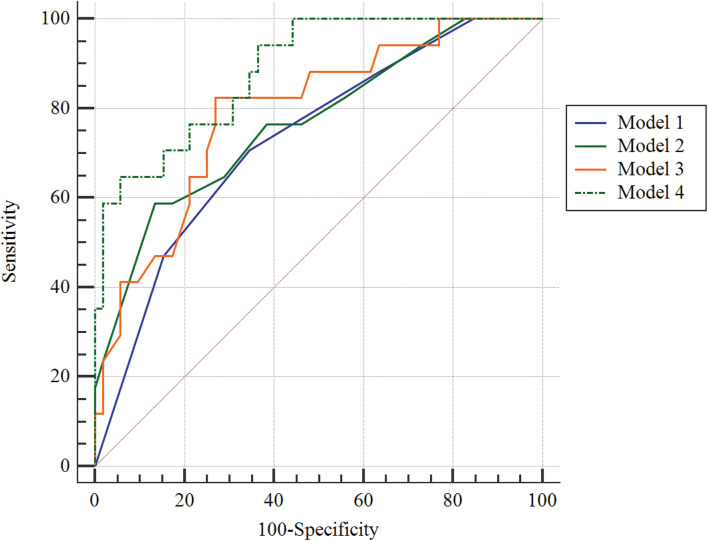
Receiver operating characteristic curve for predicting cerebral small vessel disease associated with cognitive impairment. Model 1: the total CSVD score; model 2: total DMV score; model 3: age + total CSVD score; model 4: age + total CSVD score + DMV score.

By AUROC analysis, the predictive value of the integration of patient age, total CSVD score, and DMV score (model 4) for CSVD associated with cognitive impairment was 0.885, which had a sensitivity and specificity of 64.7 and 94.2%, respectively (Figure 3). The total CSVD score (model 1) of 0.731 yielded sensitivities and specificities for predicting CSVD associated with cognitive impairment of 70.59 and 65.38%, respectively; the total DMV score (model 2) of 0.738 yielded sensitivities and specificities of 58.82 and 86.54%, respectively; and integration of patient age, total CSVD score (model 3) of 0.791 yielded sensitivities and specificities for predicting CSVD associated with cognitive impairment of 82.35 and 73.08%, respectively. After Delong’s test for pairwise comparison of AUCs between model 4 and model 1 and model 2 and model 3, the AUC improved significantly (*P* = 0.0138, 0.035, and 0.040, respectively).

## Discussion

In this study, we found that patient age and the total scores of CSVD and DMV were each independently associated with the presence or absence of cognitive impairment in patients with CSVD. The older the age, the higher the total CSVD score, and the higher the DMV score, these patients with CSVD were more likely to complain of cognitive impairment. Thus, we think that the DMV score may be a novel imaging biomarker for identifying patients with CSVD associated with cognitive impairment. The integration of patient age, total CSVD score, and DMV score for CSVD associated with cognitive impairment had high specificity identifying patients with CSVD associated with cognitive impairment.

With the development of social health services and the general improvement in living standards, the mean life expectancy of human beings has gradually increased, resulting in the gradual awareness of the effects of vascular risk factors on cognitive function and increased knowledge about the various cognitive functions and memory in the elderly. The number of patients suffering from cognitive impairment has increased rapidly, causing great damage to the quality of life of the patients themselves. Cerebrovascular disease is now the second leading cause of dementia in the elderly, after Alzheimer’s disease (Lang et al., 2018). Aging is the key risk factor for cognitive impairment. In addition, the progressive brain damage caused by CSVD is a key pathogenic factor for cognitive impairment in the elderly.

With the recent rapid development of neuroimaging, a variety of neuroimaging biomarkers have been widely recognized to be involved in the occurrence and development of cognitive impairment. The discovery of novel neuroimaging biomarkers for CSVD has gradually led to intense interest and research in the field of cognitive impairment.

Studies have indicated that the total CSVD score, which measures the overall degree of brain damage related to cognitive decline, is an evaluation standard useful for neuroimaging (Huijts et al., 2013; Staals et al., 2015). The total CSVD score shows a higher potential for reliability than an individual neuroimaging biomarker for CSVD. Our study also confirmed this conclusion, that the total CSVD score reflects the cumulative impact of different CSVD neuroimaging biomarkers on cognitive function, and that the total CSVD score has important clinical value.

The relationship between the total score of CSVD and cognitive impairment may be explained that neuroimaging biomarkers of CSVD lead to glial hyperplasia, necrosis, and demyelination of local brain tissue, thereby destroying the cortex-subcortical network of connections, and ultimately leading to impaired transmission of information within the brain (Banerjee et al., 2018; Xu et al., 2018). Other studies have reported MRI findings of large areas of microstructural damage to the white matter in normal tissues around CSVD lesions (Muñoz Maniega et al., 2019). Destruction of these microstructures will also have a serious impact on the transmission of information in the brain. Some studies have also shown that small subcortical infarcts can lead to localized atrophy of the deep cortex connected to the subcortical infarct (Lotan et al., 2019). These findings indicate that even the presence of minor CSVD imaging markers on MRI scans may be associated with prominent changes in brain structure and long-term changes in and serious effects on cognitive impairment.

The structural changes of small arteries and capillaries in the brain are significantly related to the pathogenesis of various types of dementia in the elderly (Yu et al., 2020). Nevertheless, a few studies have demonstrated that changes in the cerebral venous circulation may play an important physiological role in the pathogenesis of cognitive impairment (Fulop et al., 2019). Despite the importance of cerebral venous circulation in the pathogenesis of cognitive impairment, the role of DMVs in CSVD associated with cognitive impairment remains unclear. The results of this study indicate that the DMV score was independently correlated with cognitive impairment in patients with CSVD. According to recent studies on DMV, an increase in the DMV score or the decreased visibility of DMV in MRI scans may be related to decreased cerebral blood flow, stenosis of a vessel lumen, or even complete occlusion due to chronic hypoperfusion, hypometabolism, and changes such as PVC in walls of the veins (Fulop et al., 2019; Chen et al., 2020; Xu et al., 2020). These changes would lead to venous hypertension and retrograde venous blood flow, resulting in the disruption of the blood-brain barrier (Li et al., 2019; Wong et al., 2019) and white matter microstructure (Xu et al., 2018; Fulop et al., 2019; Muñoz Maniega et al., 2019). The cortex–subcortical connection network would then be destroyed, and the affected patients would present with cognitive impairment.

Our study has limitations. First, this study only enrolled a small sample of patients at a single center. A larger number of patients from multiple centers should be evaluated in a future study. Second, some patients were excluded because of the poor quality of their images, which might lead to bias. Third, the DMVs were assessed by direct visualization according to a qualitative scoring system. A quantitative method, such as quantitative susceptibility mapping, might be better for evaluating the DMVs. Finally, we did not follow up our study patients and evaluate the dynamic changes of DMV and progression or regression of CSVD association with the cognitive impairment, which warrants investigating in future studies.

## Conclusion

The DMV score may be a novel imaging biomarker for CSVD associated with cognitive impairment. Integrating the DMV score with age and the total CSVD score should increase the predictive value of the DMV score for CSVD associated with cognitive impairment.

## Data Availability Statement

The original contributions presented in the study are included in the article/supplementary material, further inquiries can be directed to the corresponding author/s.

## Ethics Statement

The studies involving human participants were reviewed and approved by the General Hospital of Northern Theater Command. The patients/participants provided their written informed consent to participate in this study.

## Author Contributions

ZX, FL, and YD conceived the project idea and wrote the manuscript. BY provided critical suggestions for the design of the experiments. FL, DX, HS, and JC collected the imaging and clinical data. ZX, FL, HS, JC, YD, and BY provided the imaging analysis. YD and BY supervised the project. All authors contributed to the article and approved the submitted version.

## Conflict of Interest

The authors declare that the research was conducted in the absence of any commercial or financial relationships that could be construed as a potential conflict of interest.

## Publisher’s Note

All claims expressed in this article are solely those of the authors and do not necessarily represent those of their affiliated organizations, or those of the publisher, the editors and the reviewers. Any product that may be evaluated in this article, or claim that may be made by its manufacturer, is not guaranteed or endorsed by the publisher.

## References

[B1] Amin Al Olama A. Wason J. M. S. Tuladhar A. M. Van Leijsen E. M. C. Koini M. (2020). Simple MRI score aids prediction of dementia in cerebral small vessel disease. *Neurology* 94 e1294–e1302. 10.1212/WNL.0000000000009141 32123050 PMC7274929

[B2] Banerjee G. Jang H. Kim H. J. Kim S. T. Kim J. S. Lee J. H. (2018). Total MRI small vessel disease burden correlates with cognitive performance, cortical atrophy, and network measures in a memory clinic population. *J. Alzheimers Dis.* 63 1485–1497. 10.3233/JAD-170943 29843234

[B3] Chen X. Wang J. Shan Y. Cai W. Liu S. Hu M. (2019). Cerebral small vessel disease: neuroimaging markers and clinical implication. *J. Neurol.* 266 2347–2362. 10.1007/s00415-018-9077-3 30291424

[B4] Chen X. Wei L. Wang J. Shan Y. Cai W. Men X. (2020). Decreased visible deep medullary veins is a novel imaging marker for cerebral small vessel disease. *Neurol. Sci.* 41 1497–1506. 10.1007/s10072-019-04203-9 31955350

[B5] Delavaran H. Jönsson A. C. Lövkvist H. Iwarsson S. Elmståhl S. Norrving B. (2017). Cognitive function in stroke survivors: a 10-year follow-up study. *Acta Neurol. Scand.* 136 187–194. 10.1111/ane.12709 27804110

[B6] DeLong E. R. Delong D. M. Clarke-Pearson D. L. (1988). Comparing the areas under two or more correlated receiver operating characteristic curves: a nonparametric approach. *Biometrics* 44 837–845. 10.2307/2531595 3203132

[B7] Fulop G. A. Tarantini S. Yabluchanskiy A. Molnar A. Prodan C. I. Kiss T. (2019). Role of age-related alterations of the cerebral venous circulation in the pathogenesis of vascular cognitive impairment. *Am. J. Physiol. Heart Circ. Physiol.* 316 H1124–H1140. 10.1152/ajpheart.00776.2018 30848677 PMC6580383

[B8] Huijts M. Duits A. Van Oostenbrugge R. J. Kroon A. A. De Leeuw P. W. Staals J. (2013). Accumulation of MRI markers of cerebral small vessel disease is associated with decreased cognitive function. a study in first-ever lacunar stroke and hypertensive patients. *Front. Aging Neurosci.* 5:72. 10.3389/fnagi.2013.00072 24223555 PMC3818574

[B9] Jiang Y. Wang Y. Yuan Z. Xu K. Zhang K. Zhu Z. (2019). Total cerebral small vessel disease burden is related to worse performance on the mini-mental state examination and incident dementia: a prospective 5-year follow-up. *J. Alzheimers Dis.* 69 253–262. 10.3233/JAD-181135 31006685

[B10] Lang B. Kindy M. S. Kozel F. A. Schultz S. K. Taheri S. (2018). Multi-parametric classification of vascular cognitive impairment and dementia: the impact of diverse cerebrovascular injury biomarkers. *J. Alzheimers Dis.* 62 39–60. 10.3233/JAD-170733 29439338

[B11] Li Y. Li M. Yang L. Qin W. Yang S. Yuan J. (2019). The relationship between blood-brain barrier permeability and enlarged perivascular spaces: a cross-sectional study. *Clin. Interv. Aging* 14 871–878. 10.2147/CIA.S204269 31190773 PMC6519012

[B12] Liu X. Li T. Diao S. Cai X. Kong Y. Zhang L. (2019). The global burden of cerebral small vessel disease related to neurological deficit severity and clinical outcomes of acute ischemic stroke after IV rt-PA treatment. *Neurol. Sci.* 40 1157–1166. 10.1007/s10072-019-03790-x 30830567

[B13] Lotan E. Tavor I. Barazany D. Ben-Amitay S. Hoffmann C. Tsarfaty G. (2019). Selective atrophy of the connected deepest cortical layers following small subcortical infarct. *Neurology* 92 e567–e575. 30635479 10.1212/WNL.0000000000006884

[B14] Muñoz Maniega S. Meijboom R. Chappell F. M. Valdés Hernández M. D. C. Starr J. M. Bastin M. E. (2019). Spatial gradient of microstructural changes in normal-appearing white matter in tracts affected by white matter hyperintensities in older age. *Front. Neurol.* 10:784. 10.3389/fneur.2019.00784 31404147 PMC6673707

[B15] Pavlovic A. M. Pekmezovic T. Trajkovic J. Z. Tomic G. Cvitan E. Sternic N. (2018). Increased risk of cognitive impairment and more severe brain lesions in hypertensive compared to non-hypertensive patients with cerebral small vessel disease. *J. Clin. Hypertens* 20 1260–1265. 10.1111/jch.13357 30058256 PMC8031348

[B16] Staals J. Booth T. Morris Z. Bastin M. E. Gow A. J. Corley J. (2015). Total MRI load of cerebral small vessel disease and cognitive ability in older people. *Neurobiol. Aging* 36 2806–2811. 10.1016/j.neurobiolaging.2015.06.024 26189091 PMC4706154

[B17] Wardlaw J. M. Smith E. E. Biessels G. J. Cordonnier C. Fazekas F. Frayne R. (2013). Neuroimaging standards for research into small vessel disease and its contribution to ageing and neurodegeneration. *Lancet Neurol.* 12 822–838. 10.1016/S1474-4422(13)70124-8 23867200 PMC3714437

[B18] Wong S. M. Jansen J. F. A. Zhang C. E. Hoff E. I. Staals J. Van Oostenbrugge R. J. (2019). Blood-brain barrier impairment and hypoperfusion are linked in cerebral small vessel disease. *Neurology* 92 e1669–e1677. 10.1212/WNL.0000000000007263 30867275

[B19] Xu X. Lau K. K. Wong Y. K. Mak H. K. F. Hui E. S. (2018). The effect of the total small vessel disease burden on the structural brain network. *Sci. Rep.* 8:7442. 10.1038/s41598-018-25917-4 29748646 PMC5945601

[B20] Xu Z. Li F. Wang B. Xing D. Pei Y. Yang B. (2020). New insights in addressing cerebral small vessel disease: association with the deep medullary veins. *Front. Aging Neurosci.* 12:597799. 10.3389/fnagi.2020.597799 33335483 PMC7736107

[B21] Yu C. Lu W. Qiu J. Wang F. Li J. Wang L. (2020). Alterations of the whole cerebral blood flow in patients with different total cerebral small vessel disease burden. *Front. Aging Neurosci.* 12:175. 10.3389/fnagi.2020.00175 32655393 PMC7324936

[B22] Zhang R. Zhou Y. Yan S. Zhong G. Liu C. Jiaerken Y. (2017). A brain region-based deep medullary veins visual score on susceptibility weighted imaging. *Front. Aging Neurosci.* 9:269. 10.3389/fnagi.2017.00269 28848426 PMC5550668

[B23] Zhou Y. Li Q. Zhang R. Zhang W. Yan S. (2020). Role of deep medullary veins in pathogenesis of lacunes: longitudinal observations from the CIRCLE study. *J. Cereb. Blood Flow Metab.* 40 1797–1805. 10.1177/0271678X19882918 31619117 PMC7446567

